# Supplementary material for: Downregulation of cytoplasmic DNases is implicated in cytoplasmic DNA accumulation and SASP in senescent cells
Source: Nat Commun. 2018 Mar 28;9:1249. doi: 10.1038/s41467-018-03555-8 (PMC5871854; doi:10.1038/s41467-018-03555-8)
Supplement: Supplementary file 1 — Supplementary Information(PDF 2974 kb)(PDF 3763 kb) [file 41467_2018_3555_MOESM1_ESM.pdf]

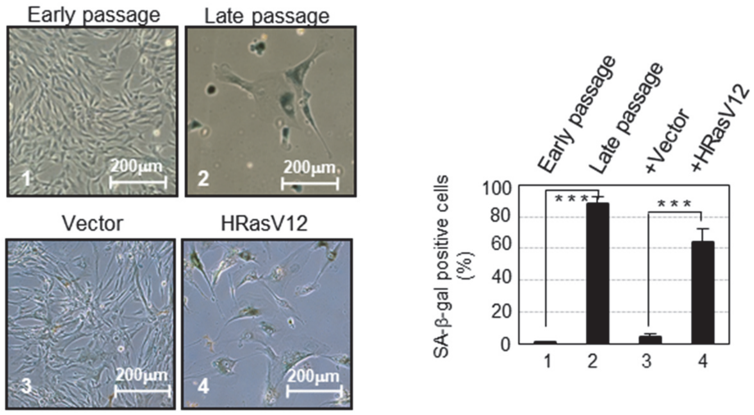

Supplementary Figure 1 | SA-β-gal activities were detected in senescent HDFs.

(**a-c**) Pre-senescent TIG-3 cells were rendered senescent by either serial passage (late passage) or ectopic expression of oncogenic *ras* (+HRasV12). These cells were then subjected to Senescence associated β-galactosidase (SA-β-gal) analysis. The histograms indicate the percentages of SA-β-gal activity positive cells. Scale bars, 200 μm. Error bars indicate mean ±standard deviation (s.d.) of triplicate measurements. ***P<0.001.

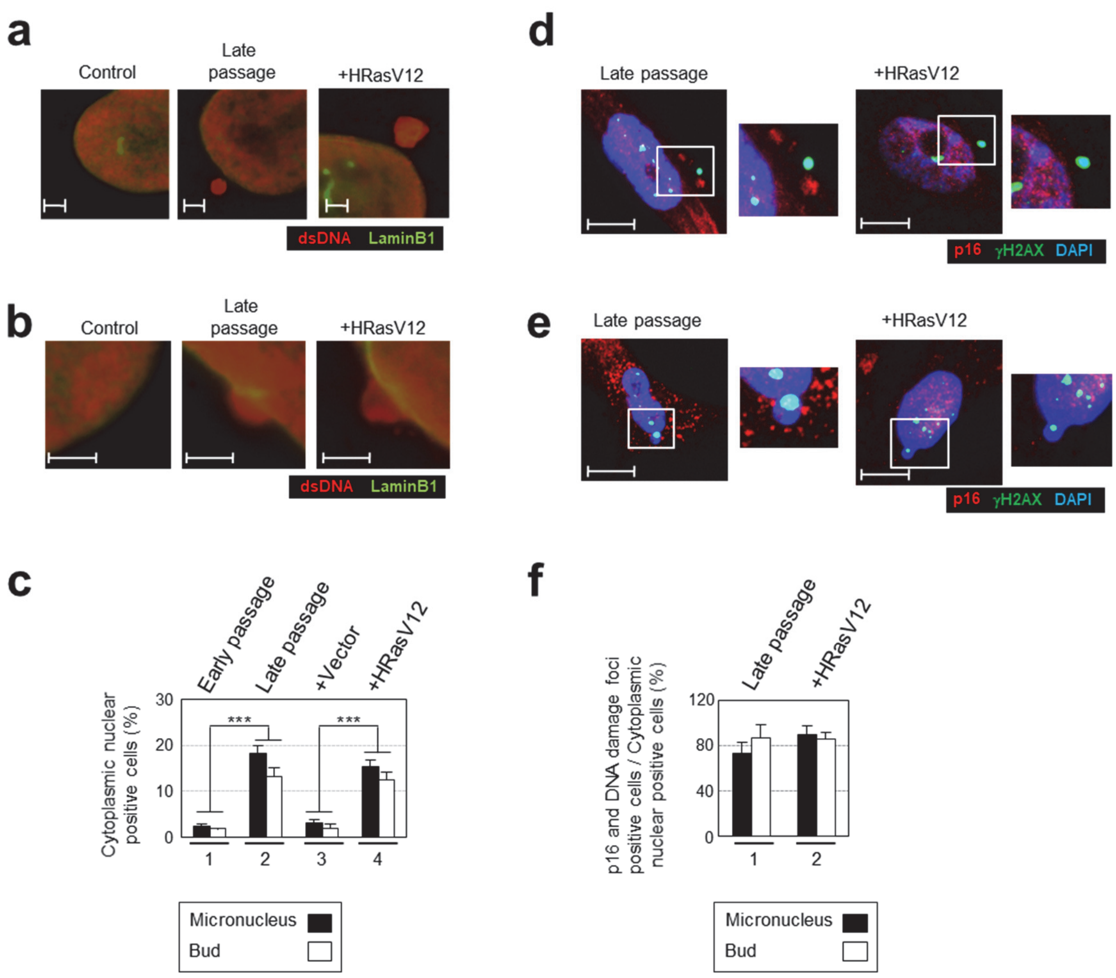

Supplementary Figure 2 | Cytoplasmic DNA accumulations were observed in senescent HDFs.

(**a-c**) Pre-senescent TIG-3 cells were rendered senescent by either serial passage (late passage) or ectopic expression of oncogenic *ras* (+HRasV12). These cells were then subjected to immunofluorescence staining for markers of dsDNA [red] and Lamin B1 [green] to detect the micronucleus (**a**) or nuclear buds (**b**) in the cytoplasm. The histograms indicate the percentages of cytoplasmic DNA positive cells for indicated markers (**c**). Scale bars, 2.5 μm. (**d-f**) Pre-senescent TIG-3 cells were rendered senescent by either serial passage (late passage) or ectopic expression of oncogenic *ras* (+HRasV12). These cells were then subjected to immunofluorescence staining for p16

[red] and markers of DNA damage foci (γ-H2AX [green]) and 40,6-diamidino-2-phenylindole (DAPI) [blue] to detect the micronucleus (**d**) or nuclear buds (**e**) in the cytoplasm. The histograms indicate the percentages of p16 and DNA damage foci positive cells in cytoplasmic DNA positive cells for indicated markers (**f**). Scale bars, 10 μm. For all graphs, error bars indicate mean $\pm$standard deviation (s.d.) of triplicate measurements. (***$P<0.001$; one-way ANOVA).

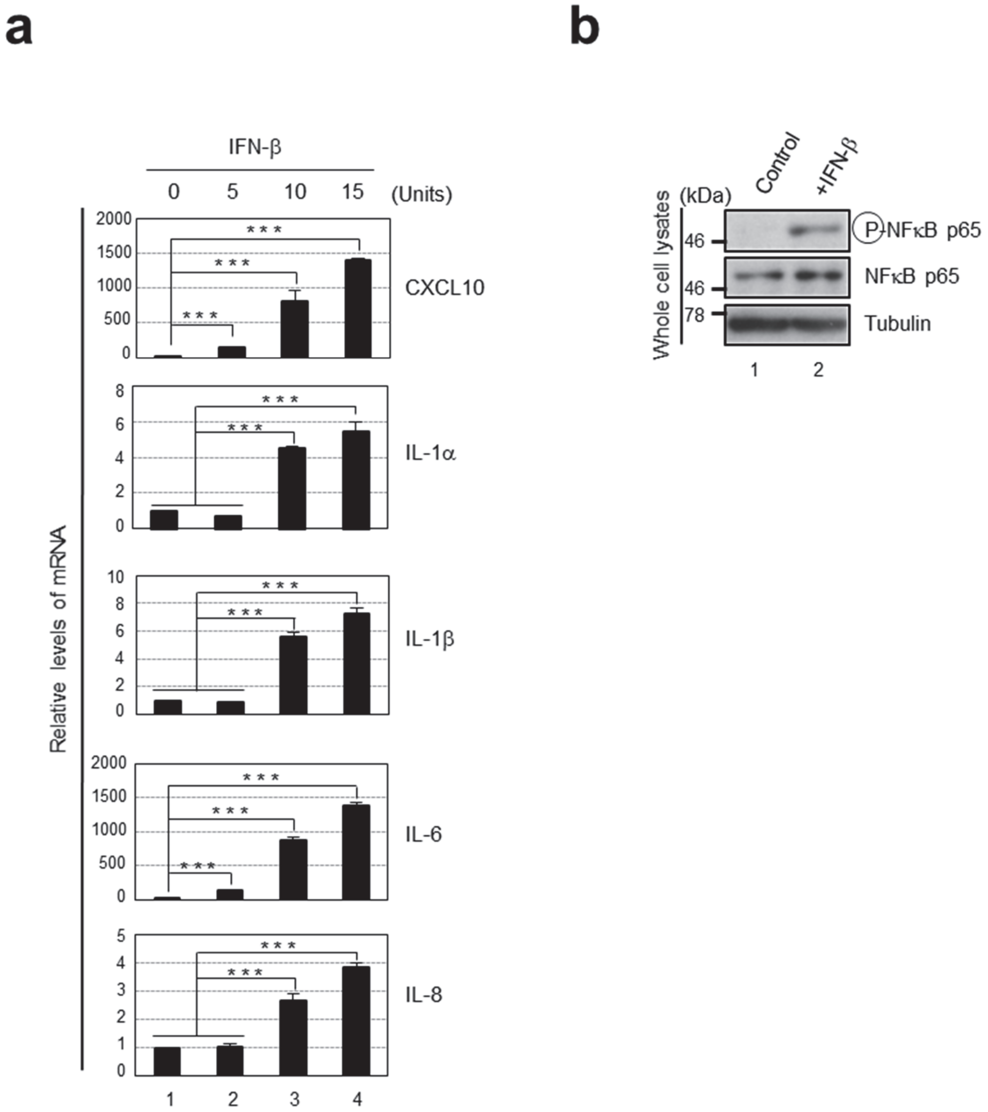

Supplementary Figure 3 | IFN-β provokes SASP in HDFs.

(a) Pre-senescent TIG-3 cells were treated with increasing amounts of recombinant IFN-β. After 12 hours, these cells were subjected to RT-qPCR analysis for detection of SASP factor gene expression shown right. **(b)** Pre-senescent TIG-3 cells were treated with 15 units/ml of recombinant IFN-β for 15 min, then subjected to western blotting using antibodies shown right. Tubulin was used as a loading control (**b**). The representative data from three independent experiments are shown. For all graphs, error bars indicate mean ±standard deviation (s.d.) of triplicate measurements. (***P<0.001; one-way ANOVA).

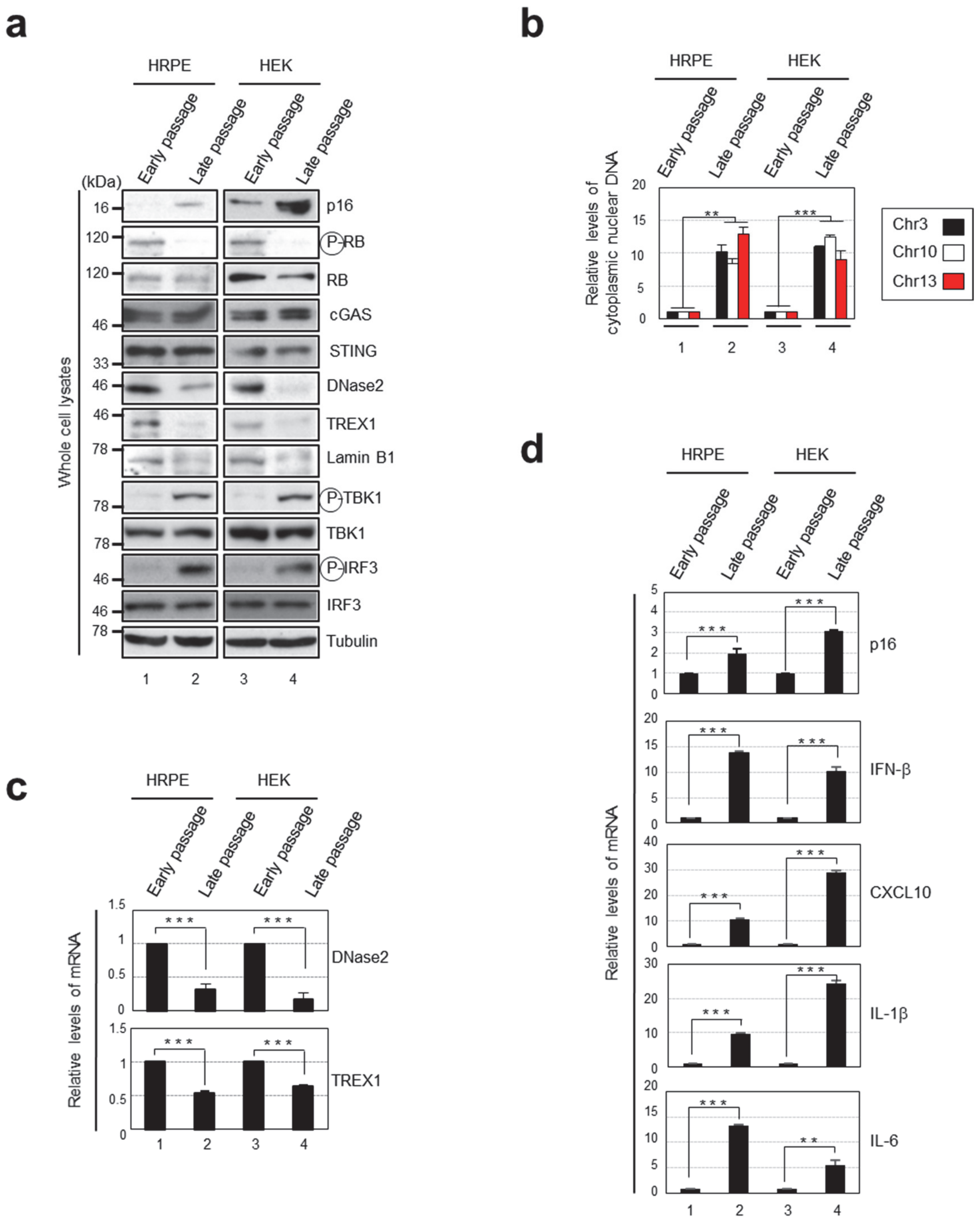

Supplementary Figure 4 | Cytoplasmic DNA sensing pathway is activated in senescent human epithelial cells.

(**a-d**) Pre-senescent primary normal human retinal pigment epithelial (HRPE) cells or pre-senescent normal human epidermal keratinocytes (HEK) were rendered senescent

by either serial passage (late passage). These cells were then subjected to western blotting using antibodies shown right (**a**), isolation of cytoplasmic fraction followed by qPCR analysis of chromosomal DNA (**b**), RT-qPCR analysis of DNase2, TREX1 (**c**) or SASP factor gene expression (**d**). The representative data from three independent experiments are shown. For all graphs, error bars indicate mean ±standard deviation (s.d.) of triplicate measurements. (**$P<0.01$. ***$P<0.001$; one-way ANOVA).

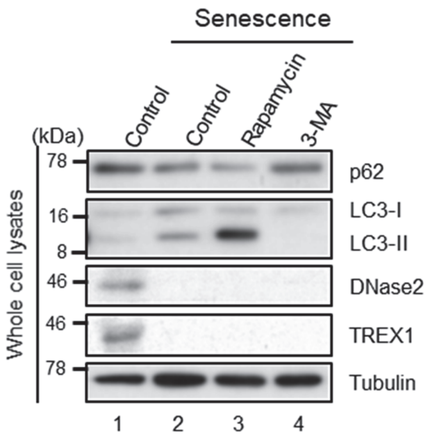

Supplementary Figure 5 | Autophagy pathway does not affect the levels of cytoplasmic DNases in cultured senescent HDFs.

Pre-senescent TIG-3 cells were rendered senescent by ectopic expression of oncogenic *ras* (+HRasV12). These cells were then subjected to treatment with 10 μM of Rapamycin or 5mM of 3-MA for 24 hours, then subjected to western blotting using antibodies shown right. Tubulin was used as a loading control.

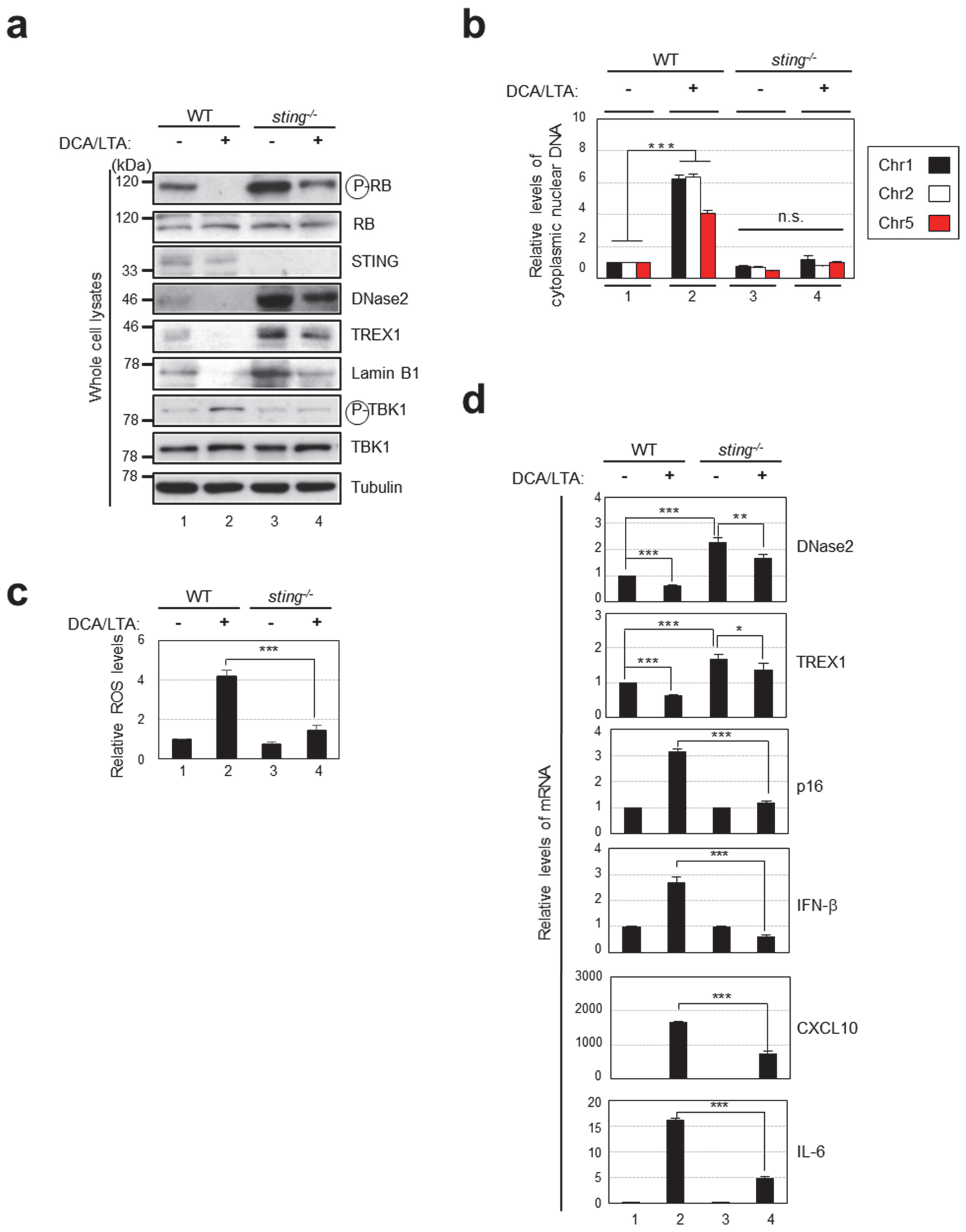

Supplementary Figure 6 | Cytoplasmic DNA sensing pathway provokes SASP in cultured senescent HSCs.

(**a-d**) Murine primary HSCs derived from WT or *sting*$^{-/-}$ mice were treated with DCA

and Lipoteichoic acid (LTA), a major constituent of the cell wall of gram-positive bacteria[12,33] for 6 days to induce cellular senescence. These cells were then subjected to western blotting using antibodies shown right (**a**), qPCR analysis of cytoplasmic nuclear DNA (**b**), analysis of intracellular ROS levels (**c**) or SASP factor gene expression (**d**). Tubulin was used as a loading control (**a**). The representative data from three independent experiments are shown. For all graphs, error bars indicate mean $\pm$standard deviation (s.d.) of triplicate measurements. (n.s. = not significant *P<0.05. **P<0.01. ***P<0.001; one-way ANOVA).

Fig. 1a

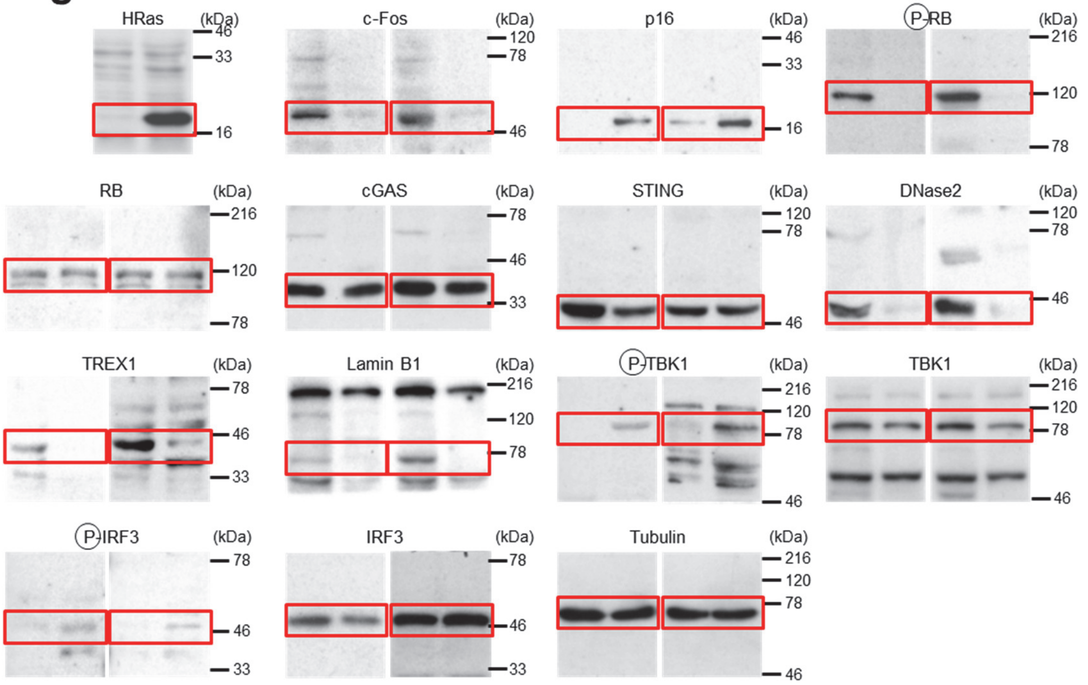

Fig. 2a

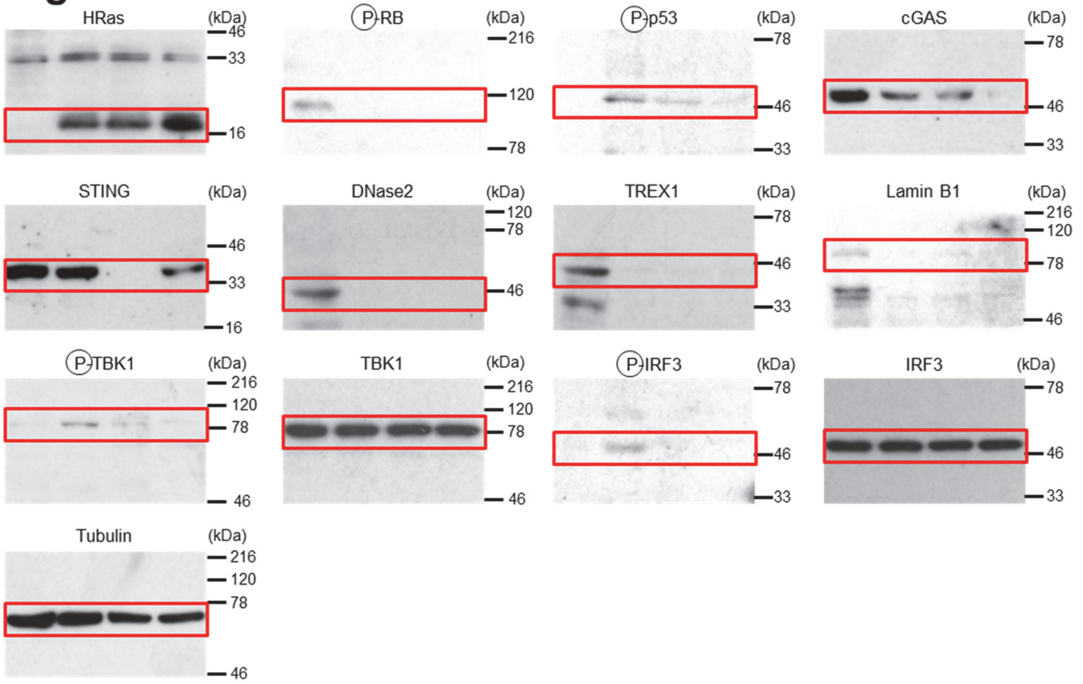

Supplementary Figure 7 | The original immunoblot images.

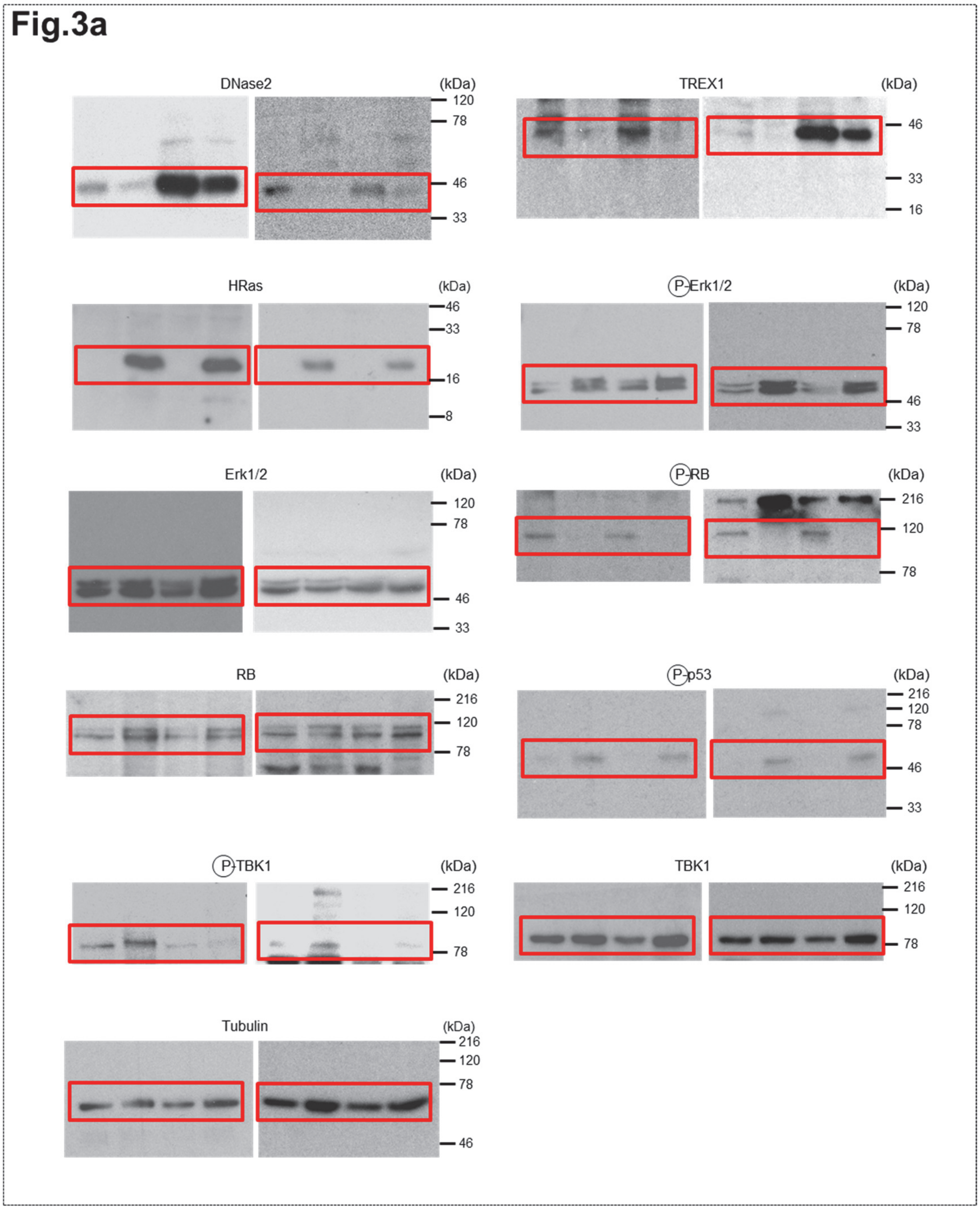

Supplementary Figure 7 | continued.

Fig. 4a

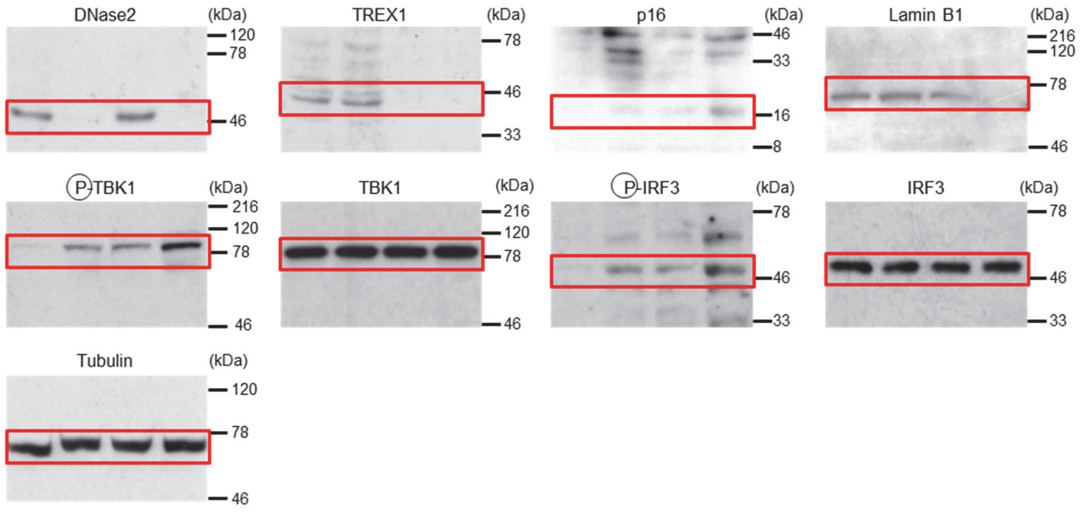

Fig. 5b

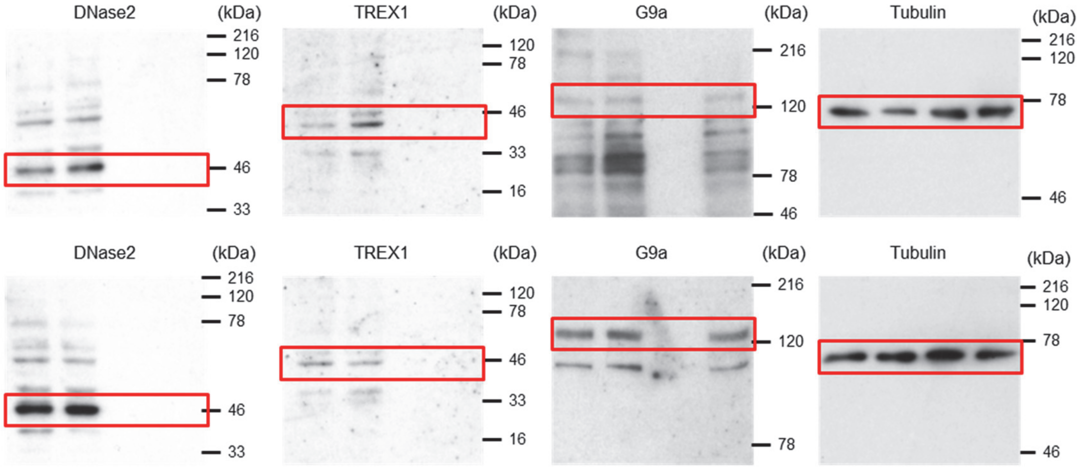

Fig. 5d

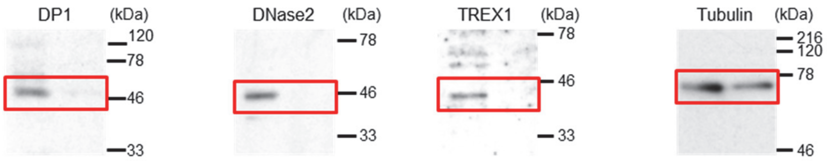

Supplementary Figure 7 | continued.

Gene		Primer	Reference No.
human GAPDH	*F*	5’-CAACTACATGGTTTACATGTTC-3’	5, 20, 30
	R	5’-GCCAGTGGACTCCACGAC-3’	
human IL-6	*F*	5’-CCAGGAGCCCAGCTATGAAC-3’	20
	R	5’-CCCAGGGAGAAGGCAACTG-3’	
human IL-8	*F*	5’-AAGGAAAACTGGGTGCAGAG-3’	20, 30
	R	5’-ATTGCATCTGGCAACCCTAC-3’	
*human IL-1*α	*F*	5’-AACCAGTGCTGCTGAAGGA-3’	20, 30
	R	5’-TTCTTAGTGCCGTGAGTTTCC-3’	
*human IL-1*β	*F*	5’-CTGTCCTGCGTGTTGAAAGA-3’	20
	R	5’-TTGGGTAATTTTTGGGATCTACA-3’	
human IFN-β	*F*	5’-AAACTCATGAGCAGTCTGCA-3’	30, 1*
	R	5’-AGGAGATCTTCAGTTTCGGAGG-3’	
human CXCL10	*F*	5’-CCAGAATCGAAGGCCATCAA-3’	30, 2*
	R	5’-CATTTCCTTGCTAACTGCTTTCAG-3’	
human p16	*F*	5’- CGAATAGTTACGGTCGGAGG-3’	66
	R	5’- TGAGAGTGGCGGGGTCG-3’	
human Chr3	*F*	5’-TCAAGTGCCACATCCTATGC-3’	30
	R	5’-ATTTTTCTAGCCAGGCACCA-3’	
human Chr10	*F*	5’-ACCTGGAAATGGCTGAAATG-3’	30
	R	5’-AAGTCCTCGCAGAGGTTTCA-3’	
human Chr13	*F*	5’-CGCCAGTGTGTGTAGCACTT-3’	30
	R	5’-TCGGCCTCTCTCAGTTCTGT-3’	
human DNase2	*F*	5’-TCGCCTTCCTGCTCTACAAT-3’	3*
	R	5’-CCCATCTTCGAGAACTGAGC-3’	
human TREX1	*F*	5’-TGCCTTCTGTGTGGATAG-3’	4*
	R	5’-AGTGTAGATGCTGCCTAG-3’	
human DP1	*F*	5’-TCCGACTCCTCACCTTGGTC-3’	39
	R	5’-CGTAGGCCCTTGCCATTCT-3’	
mouse GAPDH	*F*	5’- CAACTACATGGTCTACATGTTC-3’	66
	R	5’- CGCCAGTAGACTCCACGAC-3’	
mouse DNase2	*F*	5’-GCTCAGCTGGGGACTCTAC-3’	28

	R	5'-GGTCTGGCCGAAGGTTTGA-3'	
mouse TREX1	*F*	5'-TTCCTCAGCCACACTGCT-3'	5*
	R	5'-AGAGCTTGTCCACCACACG-3'	
mouse IL-6	*F*	5'-AGAAGGAGTGGCTAAGGACCAA-3'	66
	R	5'-AACGCACTAGGTTTGCCGAGTA-3'	
mouse IFN-β	*F*	5'- CAGCTCCAAGAAAGGACGAAC-3'	30, 6*
	R	5'- GGCAGTGTAACTCTTCTGCAT-3'	
mouse CXCL10	*F*	5'-CCAAGTGCTGCCGTCATTTTC-3'	7*
	R	5'-GGCTCGCAGGGATGATTTCAA-3'	
mouse p16	*F*	5'- GAACTCTTTCGGTCGTACCC-3'	66
	R	5'- CGAATCTGCACCGTAGTTGA-3'	
mouse Chr1	*F*	5'-GAACCGAAAGCCGTAGTTCA-3'	
	R	5'-GAACCGAAAGCCGTAGTTCA-3'	
mouse Chr2	*F*	5'- ACCCAGGACAACCACTGAAG-3'	
	R	5'- TTCCTCCCAAGAAAAAGCAA-3'	
mouse Chr5	*F*	5'-TCCGACTCCTCACCTTGGTC-3'	
	R	5'-CGTAGGCCCTTGCCATTCT-3'	

*Supplementary references

Supplementary Table 1 | List of Quantitative Real-Time PCR primers

Supplementary References

1. Orzalli, M.H. *et al.* cGAS-mediated stabilization of IFI16 promotes innate signaling during herpes simplex virus infection. *Proc. Natl. Acad. Sci. U. S. A.* **112**, E1773-1781 (2015).
2. Khan, S. *et al.* Differential gene expression of chemokines in KRAS and BRAF mutated colorectal cell lines: role of cytokines. *World J. Gastroenterol.* **20**, 2979-2994 (2014).
3. Fischer, H. *et al.* DNase1L2 degrades nuclear DNA during corneocyte formation. *J. Invest. Dermatol.* **127**, 24-30 (2007).
4. Zhang, Y. *et al.* The DNA sensor, cyclic GMP-AMP synthase, is essential for induction of IFN-beta during Chlamydia trachomatis infection. *J. Immunol.* **193**, 2394-2404 (2014).
5. Pereira-Lopes, S. *et al.* The exonuclease Trex1 restrains macrophage proinflammatory activation. *J. Immunol.* **191**, 6128-6135 (2013).
6. Turer, E.E. *et al.* Homeostatic MyD88-dependent signals cause lethal inflamMation in the absence of A20. *J. Exp. Med.* **205**, 451-464 (2008).
7. Morales, A.J. *et al.* A type I IFN-dependent DNA damage response regulates the genetic program and inflammasome activation in macrophages. *Elife* **6** (2017).
